# New Insight into the Composition of Wheat Seed Microbiota

**DOI:** 10.3390/ijms21134634

**Published:** 2020-06-30

**Authors:** Agnieszka Kuźniar, Kinga Włodarczyk, Jarosław Grządziel, Małgorzata Woźniak, Karolina Furtak, Anna Gałązka, Ewa Dziadczyk, Ewa Skórzyńska-Polit, Agnieszka Wolińska

**Affiliations:** 1 Department of Biology and Biotechnology of Microorganisms, The John Paul II Catholic University of Lublin, Konstantynów St. 1 I, 20-708 Lublin, Poland; kingawlodarczyk@kul.lublin.pl (K.W.); agnieszka.wolinska@kul.pl (A.W.); 2 Department of Agricultural Microbiology, Institute of Soil Science and Plant Cultivation, Czartoryskich St. 8, 24-100 Puławy, Poland; jgrzadziel@iung.pulawy.pl (J.G.); m.wozniak@iung.pulawy.pl (M.W.); kfurtak@iung.pulawy.pl (K.F.); agalazka@iung.pulawy.pl (A.G.); 3 Department of Plant Physiology and Biotechnology, The John Paul II Catholic University of Lublin, Konstantynów St. 1 I, 20-708 Lublin, Poland; ewa.dziadczyk@kul.pl (E.D.); eskorzynska@kul.lublin.pl (E.S.-P.)

**Keywords:** endophytes, seeds, embryos, endosperm, wheat, core microbiome, NGS

## Abstract

Endophytes are associated with host plants throughout their life history from seed germination to fruit development. One of the most important plant organs colonized by endophytic microbiota is the seed. The aim of this study was to determine the structure of the seed core microbiome inhabiting the endosperms and embryos of eight wheat cultivars with the use of a culture-independent technique. The seeds of *Triticum aestivum* L. cv. Hondia, Wilejka, STH, Opcja, Tybalt, Euforia and *Triticum spelta* L. cv. Rokosz and Schwabencorn (producer: Plant Breeding Strzelce Sp. z o.o. Group IHAR) were studied. Rokosz and Hondia were cultured in vitro and in vivo to identify obligatory bacterial endophytes. A restrictive analysis of reads originating from the in vitro plants has demonstrated that the bacterial genera *Paenibacillus* and *Propionibacterium* inhabiting Rokosz and Hondia plants have a status of obligatory microorganisms. Greater biodiversity of seed-borne endophytes was found in the seed endosperms than in the embryos. The multiple comparison analysis of the OTU abundance indicated that the seed part significantly influenced the relative abundance. The seed-born microbiome is not statistically significantly dependent on the wheat cultivars; however, it cannot be claimed that every wheat seed is the same.

## 1. Introduction

Plants harbor multiple endophytic taxa (bacteria and fungi) mainly exerting a positive effect on the host, i.e., the production of metabolites, drought tolerance, and resistance to pathogens [1,2,3,4,5]. Importantly, the endophytic microbiota is associated with the host plant throughout its whole life history, from seed germination to fruit development [2,4]. Seeds, which are composed of the three main compartments—the embryo, endosperm, and the seed coat—seem to be one of the most important plant organs colonized by endophytic microbiota [3]. It should be underlined that early growth stages are critical for all microbiota, and plants should benefit from seed-stored bacteria in terms of proper ecological function [2,4,6,7]. As demonstrated by Geisen et al. [8], seeds are extremely important in the life cycle of Spermatophytes, as they can persist in a dormancy state even for a long time until growth conditions become suitable for them to develop into a new plant. This feature (the ability to reside in a seed and adapt to unfavorable conditions) is one of the special characteristics of seed-borne endophytes only, as those originating from other plant tissues do not have such properties [4]. Other unique features of seed-borne endophytes include cell motility and phytase activity, which allow them to enter seeds before they harden [4]. Some seed-borne microbiota can promote the germination process by the release of seed dormancy via cytokinin production [4,9]. Furthermore, it has been emphasized that the seed microbiota represents a starting point for the community assembly of the new seedling microbiome and simultaneously an endpoint for community assembly within the seed [3,4]. Therefore, seed-associated microbiota can be referred to as seed-borne or seed-transmitted microorganisms [3,4,6]. Robinson et al. [2] and Shade et al. [3] noted that the transmission of microorganisms via seeds should be considered as a relevant factor influencing the plant microbiome structure, and ultimately, plant productivity. However, this link has been less frequently studied and is still underestimated [6,7].

Based on the lifestyles of endophytes, Hardoim et al. [1] proposed their classification into obligate—microorganisms requiring plant tissues to complete their life cycle, opportunistic—microbiota that mainly thrive outside plant tissues (epiphytes) and seldom enter in the endosphere, and facultative—considered as an intermediate group between the two other extremes, comprising a vast majority of endophytes. The endophytic microbiota identified in the present study was classified according to the lifestyle. Since many endophytes originate from the rhizosphere environment, which attracts microorganisms due to the presence of root exudates and rhizodeposits [10,11], rhizospheric soil from *T. aestivum* L. cv. Hondia and *T. spelta* L. cv. Rokosz cultivation was investigated. Shade et al. [3] suggested that assembly patterns should be assessed for different plant species by high-throughput sequencing, which will allow researchers to define a core microbiota associated with specific seed genotypes.

Previous studies demonstrated different endophytes colonizing the different parts of seeds of different plants [3], i.e., *Fusarium culmorum* and *Epichloe typhina* were found in embryos [3], *Clavibacter michiganensis* and *Verticillium dahliae* were present in the endosperm of tomato [12], whereas *Bacillus* and *Alternaria alternata* colonized the seed coat of grapevine [10]. To our knowledge, *Oryza sativa* [13,14,15] and *Zea mays* [16,17,18] are the best recognized plants in terms of the endophytic microbiota isolated from the seeds. In wheat seeds (*Triticum aestivum* L. and *Triticum spelta* L.), Kuźniar et al. [5] identified the presence of *Acinetobacter, Pantoea, Paenibacillus, Pseudomonas, Stenotrophomonas, Paracoccus*, and *Flavobacterium*. The presence of *Bacillus, Paenibacillus*, and *Pantoea* in spring wheat endosperms were also shown by Herrera et al. [19]. Robinson et al. [2] detected *Erwinia* and *Paenibacillus* in *T. aestivum* L. cv. Hereward. However, the endophytic microbiota structure in wheat seeds (separately in the embryo and endosperm) has not been fully explored yet. Moreover, Robinson et al. [2] hypothesized that the bacterial load is carried in the wheat seed coat, crease tissue, and endosperm, but is absent in wheat embryos. However, their results were based on the culture-dependent method. In the present study, we performed a culture-independent analysis of the seeds of eight winter wheat cultivars: Euforia, Hondia, Opcja, Rokosz, Schwabencorn, STH, Tybalt, and Wilejka, in order to verify the previous findings [2].

We hypothesized in this study that the microbial seed load differs between the wheat cultivars and depends on the seed part (embryo, endosperm); however, it is possible to determine the core endophytic microbiome colonizing a majority of wheat seeds. Therefore, the main goal of this experiment was to determine the structure of the seed core microbiome inhabiting the endosperms and embryos of eight wheat cultivars with the use of a culture-independent technique. Moreover, in two of the selected specimens—*Triticum spelta* cv. Rokosz and *Triticum aestivum* cv. Hondia—the in vitro experiments were followed by an analysis of rhizospheric soil and plant fragments (leaves, roots, coleoptiles) to evidence that the same endophytes were present not only in the seeds but are also transmitted to different plant fragments and rhizospheric soil. Such an approach provides a more comprehensive insight into the structure of endophytes colonizing wheat tissues and is a new element of research because, as mentioned above, the link between the seed and soil/in vitro microbiome remains unrecognized.

## 2. Results

### 2.1. Seed-Borne Microbiome of Endosperms and Embryos of Different Wheat Cultivars

**Figure 1:**
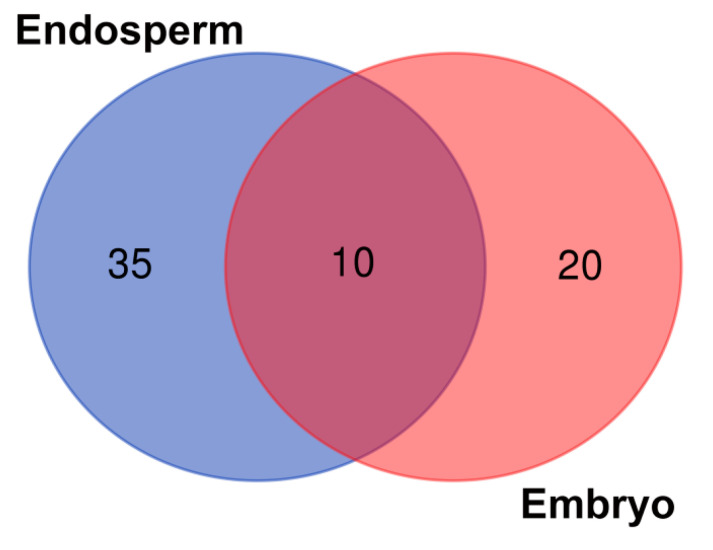
Seed-borne core microbiome of endosperms and embryos of eight wheat cultivars. The numbers included in the Venn diagram refer to the occurrence of the identified genera representing the unique and shared seed-borne core microbiome of the endosperms and embryos of the eight wheat cultivars.

The composition of the seed core microbiome present in the endosperms and embryos of eight wheat cultivars is presented in Figure 1 and Table 1. Greater biodiversity of seed-borne endophytes was found in the seed endosperms (35) rather than in the embryos (20). Only 10 endophytic genera seemed to be common for all the studied seeds. 

Moreover, although the embryos and endosperms of the wheat seeds were inhabited by a low number of bacteria, it is possible to assume that the seeds were not sterile and their embryos were colonized by the endophytes. The representatives of the bacterial genera shown in Figure 1 are summarized in Table 1. In turn, detailed information about the composition of the seed-borne microbiome present in the endosperms and embryos of the eight wheat cultivars is presented in Tables S1 and S2. 

**Figure 2:**
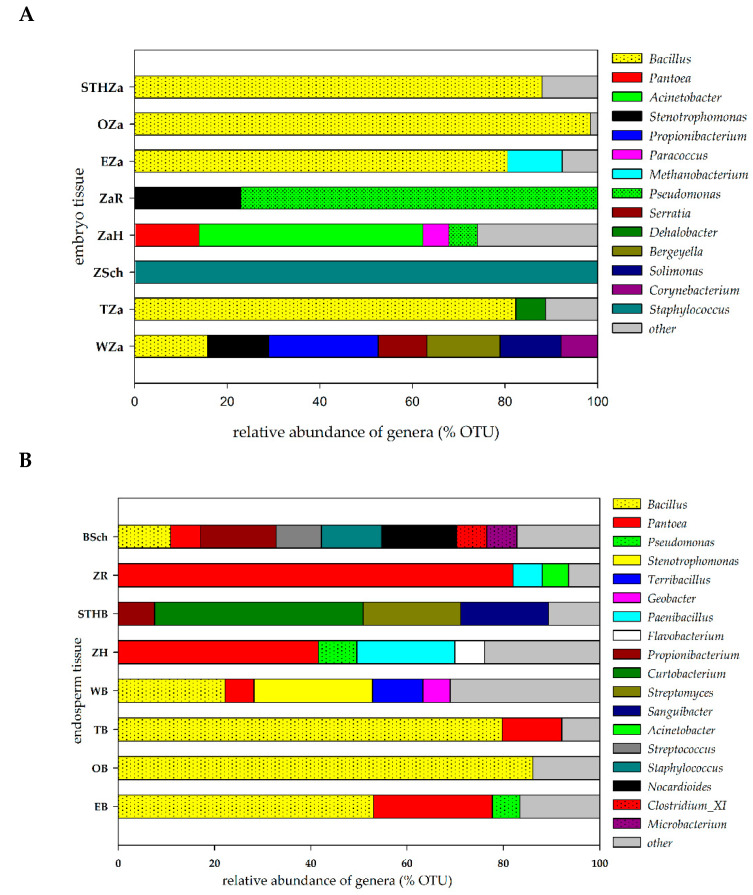
Relative abundance of the potential endophytic bacteria in seed tissue: (**A**)—embryo tissue, (**B**)—endosperm tissue. The group “other” was created based on a relative abundance lower than 5%. The overall relative abundance and number of OTUs are shown in the Suplementary material (Table S3). The abbreviations are the following: STHZa/STHB—STH embryo/endosperm, OZa/OB—Opcja embryo/endosperm, EZa/EB—Euforia embryo/endosperm, ZaR/ZR—Rokosz embryo/endosperm, ZaH/ZH—Hondia embryo/endosperm, ZSch/BSch—Schwabencorn embryo/endosperm, Tza/TB—Tybalt embryo/endosperm, WZa/WB—Wilejka embryo/endosperm.

The potential endophytic microorganisms identified in the seed tissues of the studied varieties (the embryo and endosperm) were assumed to represent relative abundance in order to construct Figure 2. The number of OTUs is shown in the Supplementary material (Table S3). We identified 41 bacterial OTUs in the embryo tissues originating from the seeds of the eight cultivars. Our results indicated that the genus *Bacillus* was dominant and accounted for ca. 75% of seeds. Figure 2A showed that the embroy tissue from the Wilejka and Hondia varieties were the most diverse in terms of bacterial genera. The least abundant OTUs were detected in the embryo tissue from the Schwabencorn cultivar (Figure 2A).

The endosperm tissue of the studied varieties showed a much higher number and diversity of OTUs (Figure 2B). *Bacillus* was the most common bacterial genus in the endosperm. The presence of this genus was reported in five wheat varieties (62.5%). Our results indicate the presence of the genus *Pantoea* in 87.5% of the analyzed seeds. According to Figure 2B, the highest diversity was observed in the endosperm isolated from Schwabencorn (eight different bacterial genera) and Wilejka (five different bacterial genera) cultivars. The alpha diversity analysis was based on the OTU table. The results of this analysis are presented in the Suplementary material (Table S3). Generally, the biodiversity (H’) index reached higher values for the endosperm tissue in relation to embryo tissue. The highest values were noted for Wilejka and Schwabencorn endosperm followed by similarly biodiverse Hondia, STH and Euforia endosperm (Table S3). Samples were characterized by the values of Simpson index (D) in the range of 0.1359–0.9689.

**Figure 3:**
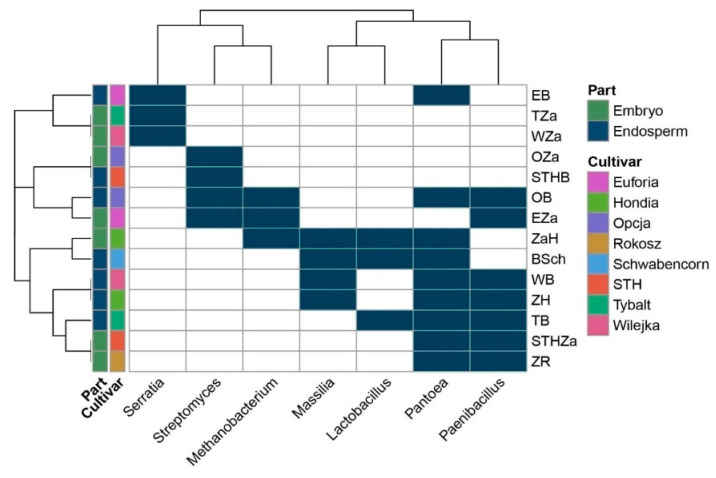
Obligatory endophytic microbiome—heat map illustrating the presence of the selected genera in the embryos and endosperms of the eight wheat cultivars. The abbreviations are the following: STHZa/STHB—STH embryo/endosperm, OZa/OB—Opcja embryo/endosperm, EZa/EB—Euforia embryo/endosperm, ZaR/ZR—Rokosz embryo/endosperm, ZaH/ZH—Hondia embryo/endosperm, ZSch/BSch—Schwabencorn embryo/endosperm, Tza/TB—Tybalt embryo/endosperm, WZa/WB—Wilejka embryo/endosperm.

The Venn diagram (Figure 1) was constructed based on all the bacteria detected in this study (even those represented by low counts, i.e., one read). The heatmap (Figure 3) included: (1) the bacteria that were present in at least 10 reads and (2) bacteria that were present in at least three samples. Consequently, it is worth mentioning that the seven bacterial genera indicated in the heatmap have the status of obligatory microorganisms inhabiting wheat seeds.

In a selected group of bacteria, the *Pantoea* and *Paenibacillus* were the dominant genera in the studied seed material. The presence of *Pantoea* was confirmed in the endosperms of the Euforia, Opcja, Schwabencorn, Wilejka, Hondia, and the Tybalt cultivars and in the embryos of Hondia, STH, and Rokosz (Figure 3). The data prove that the endosperm can be a more favorable environment for *Pantoea* colonization than the embryo. A similar trend was noted in *Paenibacillus*, whose representatives were detected in the endosperms of Opcja, Wilejka, Hondia, and Tybalt seeds more often than in the embryos of Euforia, STH, and Rokosz. *Streptomyces* and *Massilla* (Figure 3) were subdominants in the wheat seeds. The presence of *Streptomyces* was confirmed in the embryos of Opcja and Euforia cv. and in the endosperms of STH and Opcja, whereas *Masilla* colonized the endosperms rather than the embryo in Schwabencorn, Wilejka, and Hondia. The presence of *Masilla* in the embryo was noted only in the case of Hondia. The *Serratia*, *Methanobacterium*, and *Lactobacillus* genera occurred sporadically in the wheat seeds. The presence of *Serratia* was noted in the embryos of Wilejka and Tybalt cv. and in the endosperm of Euforia (Figure 3).

*Methanobacterium* representatives were present in the embryos of Euforia and Hondia and in the endosperm of Opcja, whilst the genus *Lactobacillus* was identified in the endosperms of Tybalt and Schwabencorn and in the embryo of Hondia (Figure 3). 

To sum up, it is possible to conclude that although the endophytes prefer inhabiting the endosperm, they are also able to colonize wheat embryos; hence, they are further transmitted during wheat growth. 

**Figure 4:**
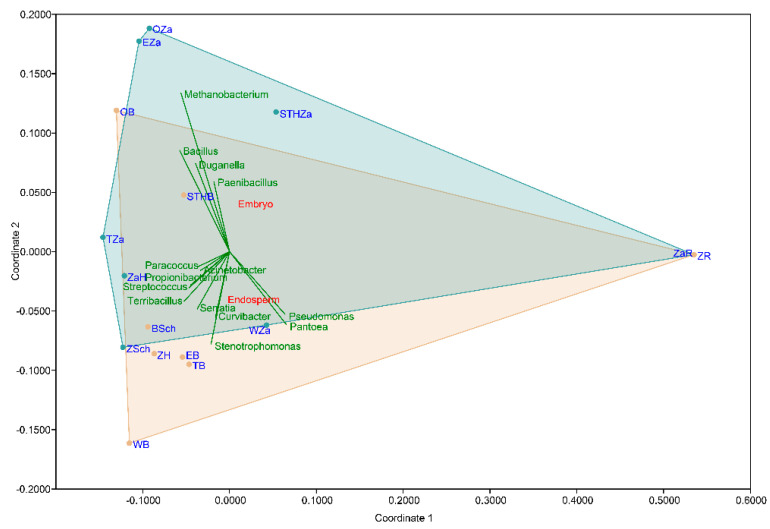
Non-metric multidimensional scaling (NMDS) visualizations of the beta diversity analysis using the Bray–Curtis metric in the studied seed samples. Each point in the NMDS plot represents the seed microbiota, and the colored boxes visualize the endosperm and embryo tissue.

The non-metric multidimensional scaling (NMDS) plot on the Bray-Curtis distance matrix (Figure 4) did not clearly distinguish the clusters based on the wheat varieties (*p* = 0.6591; F = 0.777). In the NMDS plot, each point represents the seed microbiota and the colored boxes visualize the endosperm and embryo tissue. The multiple comparison analysis on the OTU abundance and genera abundance indicated that the part of the seed significantly influenced the relative abundance (*p* = 0.0158; F = 5.335). 

### 2.2. In Vitro Experiment—Endophytic Microbiome Colonizing the Roots and Leaves of Hondia and Rokosz Cultivars

**Figure 5:**
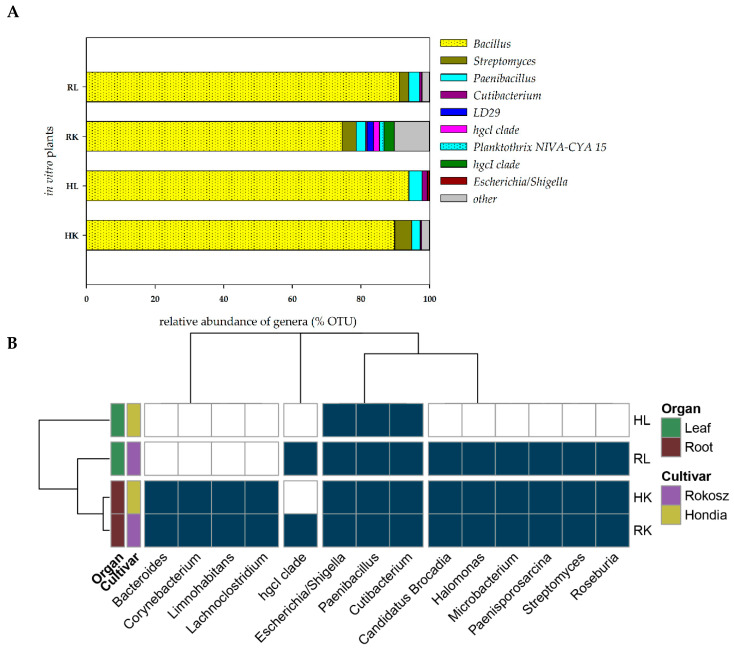
(**A**)—Relative abundance of the potential endophytic bacteria of the in vitro plant tissue. The group “other” was created based on a relative abundance lower than 1%. The overall relative abundance and number of OTUs are shown in the Suplementary material (Table S4). (**B**)—Obligatory endophytic microbiome—heat map illustrating the presence of the selected bacterial genera in the leaves and roots of Rokosz and Hondia cv. cultivated in vitro (HL—Hondia leaf; RL—Rokosz leaf; HK—Hondia root; RK—Rokosz root).

The seeds of two cultivars, i.e., *T. aestivum* L. cv. Hondia and *T. spelta* L. cv. Rokosz, were selected for the isolation of embryos and in vitro culturing. After plating on the in vitro medium, sterilized embryos were grown until stage BBCH13. DNA was isolated from the leaves and roots of the wheat specimens with no symptoms of bacterial or fungal infection during in vitro culturing. The heat map illustrating the presence of the selected bacterial genera (present in at least two of four samples with a total occurrence in all four samples of at least 0.2%) in the leaves and roots of Rokosz and Hondia cultivated in vitro is shown in Figure 5. 

It was found that the endophytes were present both in the roots and in the leaves in the in vitro conditions. This fact undeniably proves that even sterilized wheat embryos growing on in vitro media should not be considered as a sterile niche, as they are inhabited by the seed-borne endophytic microbiome, which is transported into the roots and leaves even in in vitro conditions (Figure 5). We evidenced that the roots of both wheat specimens were more preferable organs for the endophytes than the leaves, where the biodiversity was limited. Interestingly, the same representatives of the 13 bacterial genera were identified in the roots of both studied cultivars. These included *Bacteroides*, *Corynebacterium*, *Limnohabitants*, *Lachnoclostridium*, *Escherichia/Shigella*, *Paenibacillus*, *Cutibacterium*, *Candidatus Brocardia*, *Halomonas*, *Microbacterium*, *Paenisporosarcina*, *Streptomyces*, and *Roseburia* (classified as other on the Figure 4A). The biodiversity of the endophytes was higher in the leaves of Rokosz cv. than in Hondia, where only three genera (*Escherichia/Shigella*, *Paenibacillus*, *Cutibacterium*) were identified. 

In the Rokosz leaves, the following nine genera were detected: *Escherichia/Shigella*, *Paenibacillus*, *Cutibacterium*, *Candidatus Brocardia*, *Halomonas*, *Microbacterium*, *Paenisporosarcina*, *Streptomyces*, and *Roseburia*.

The *Bacillus* species were the most common bacterial genus in the roots and in the leaves of both cultivars of wheat in the in vitro experiment (more than 90% in the Hondia and Rokosz leaves, 89.83% in the Hondia roots, and 74.49% in the Rokosz roots). Bacteria belonging to the genera *Paenibacillus* and *Cutibacterium* were present in all the parts of the studied cultivars. No representatives of the genus *Streptomyces* as endophytes were found only in the Hondia leaves. The OTU results of this analysis are presented in the Suplementary material (Table S4). 

### 2.3. Comparison of Shared and Unique Endophytic Genera Inhabiting Three Niches: Rhizospheric Soil and the Leaves and Roots of T. aestivum L. Hondia—Field and In Vitro Experiments

**Figure 6:**
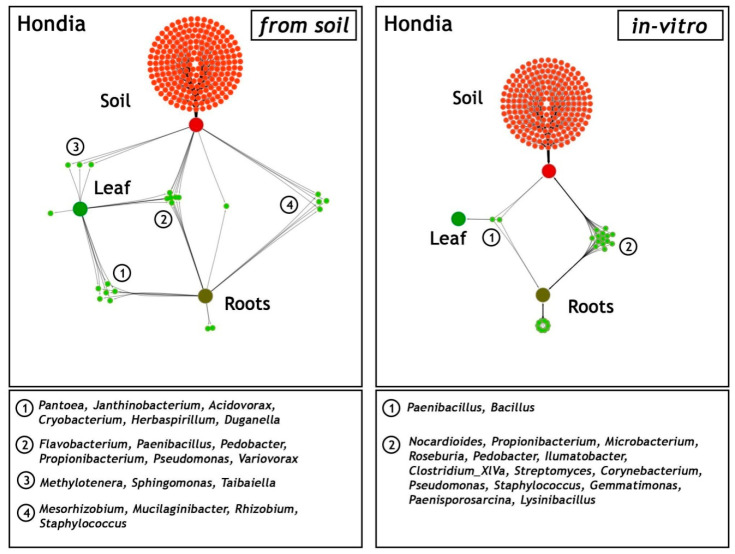
Network-like Venn diagrams of the shared and unique genera in three habitats: soil from the rhizosphere zone and the leaves and roots of *Triticum aestivum* L. cv. Hondia from the field (soil) and the in vitro experiment. The green lines represent the occurrence (single line) or co-occurrence (multiple lines) of the bacteria (species) in each sample.

A network-like Venn diagram of the common and unique endophytic communities present in the rhizosphere zone (soil) and in the leaves and roots of *T. aestivum* L. cv. ‘Hondia’ originating from the field and the in vitro experiment variants is shown in Figure 6. 

In the field (soil) variant, the bacterial genera *Pantoea*, *Janthinobacterium*, *Acidovorax*, *Cryobacterium*, *Herbaspirillum*, and *Duganella* contributed to the majority of the sequences identified in both the roots and the leaves of Hondia. Six other endophyte genera, i.e., *Flavobacterium*, *Paenibacillus*, *Pedobacter*, *Propionibacterium*, *Pseudomonas*, and *Variovorax* were detected in three habitats: the Hondia leaves, roots, and the rhizosphere soil. Relatively low diversity of the endophytic microbiome was noted in the *T. aestivum* leaves, where the presence of only three genera: *Methylotenera*, *Sphingomonas*, and *Tabaiella* was confirmed. The genera *Mesorhizobium*, *Mucilaginibacter*, *Rhizobium*, and *Staphylococcus* were classified as dominant unique representatives in two niches: and endosphere roots and the rhizospheric soil of Hondia from the field variant. 

A completely different trend in the endophytic microbiome structure was found in the endosphere of the Hondia cv. originating from the in vitro experiment. In the leaves of Hondia grown in vitro, only two shared endophytes, i.e., *Bacillus* (93.98% of sequences) and *Paenibacillus* (6.02%), were detected. The bacterial structure evidenced in two habitats (rhizospheric soil and roots from the in vitro variant) is surprising, as 16 general prokaryotes that are unique to these two ecological niches were identified (Figure 6). However, it should be mentioned that each of the identified bacterial genera was included in Figure 4, even those occurring sporadically (<0.05%). Other genera, i.e., *Streptomyces* (4.96%), *Microbacterium*(0.12%), *Paenisporosarcina* (0.13%), and *Corynebacterium* (0.13%), were detected as obligatory endophytic microbiome, characteristic for the leaves and roots of both cultivars (Hondia, Rokosz) growing in vitro (Figure 3). In this context, the other 12 genera: *Nocardioides* (0.03%), *Propionibacterium* (0.03%), *Roseburia* (0.26%), *Pedobacter* (0.04%), *Ilumatobacter* (0.02%), *Clostridium* (0.02%), *Pseudomonas* (0.05%), *Staphylococcus* (0.25%), *Gemmatimonas* (0.02%), and *Lysinibacterium* (0.04%) can be classified as the occasionally accompanying microbiome of Hondia cv. in vitro. The in vitro presence of these genera requires additional studies.

### 2.4. Comparison of Shared and Unique Endophytic Genera Inhabiting Three Niches: Rhizospheric Soil and the Leaves and Roots of T. spelta L. Rokosz—Field and In Vitro Experiments

**Figure 7:**
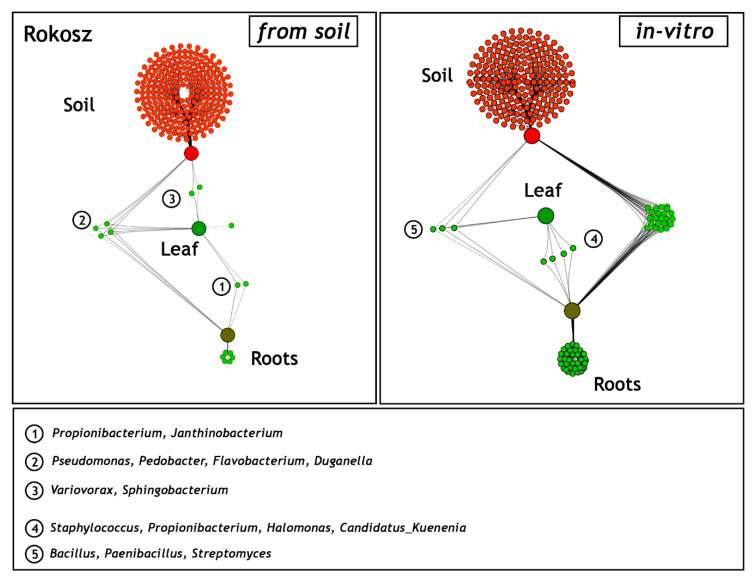
Network-like Venn diagrams of the shared and unique genera in the three habitats: soil from the rhizosphere zone and the leaves and roots of *Triticum spelta* L. cv. Rokosz from the field (soil) and the in vitro experiment. The green lines represent the occurrence (single line) or the co-occurrence (multiple lines) of the bacteria (species) in each sample.

As in the case of the cultivar described above, a network-like Venn diagram of the common and unique endophytic communities in the rhizosphere zone (soil), leaves, and roots of *T. spelta* L. cv. Rokosz originating from the field and in vitro experiments was constructed and presented in Figure 7.

Two shared bacterial genera (*Propionibacterium* and *Janthinobacterium*) were identified in the leaves and roots of Rokosz grown in the soil variant. Four other genera (*Pseudomonas*, *Pedobacter*, *Flavobacterium*, and *Duganella*) were present in the three niches: rhizospheric soil, leaves, and roots. The relatively low abundance of a network-like endophytic microbiome (*Variovorax*, *Sphingobacterium*) was found in the leaves and rhizosphere zone of Rokosz. 

The generated network-like Venn diagrams of the endophytic bacteria inhabiting the roots and leaves of *T. spelta* grown in vitro were very interesting (Figure 6). The bacteria were represented by *Staphylococcus* (0.9%), *Propionibacterium* (0.51%), *Halomonas* (0.43%), and *Candidatus Kuenenia* (0.31%). Furthermore, we observed that the *Propionibacterium* genus was shared by both the endosphere of the leaves and the roots from the in vitro and field experiments. *Bacillus* (91.3%), *Paenibacillus* (5.58%), and *Streptomyces* (3.12%) were evidenced as a component of the endophytic microbiome inhabiting the three ecological niches: the rhizosphere zone from the field experiment and the endosphere of the leaves and the roots of the in vitro plants (Figure 7).

## 3. Discussion

Seeds, like no other plant organ, provide information about the origin of plant microbiota and the mechanisms in which interactions with seed-associated microbes may improve plant growth [2,7]. It is beneficial for an endophyte to establish the successful colonization of the host plant at a very early stage at minimum competition [2]. Since seed-borne microbiota comprises initial microbial colonizers of emerging seedlings before the recruitment of microbes from the surrounding environment, it plays important roles in the assembly and function of the plant microbiome [7]. Moreover, seed endophytes are of particular interest, as they are transmitted from generation to generation [4,6]. Importantly, the bacterial identities and location in wheat seeds are unknown [2]. Therefore, any new knowledge of seed-borne endophytes is extremely desirable. 

Here, we identified the bacteria that were present in the seeds of the eight wheat cultivars (Table 1, Figure 3) and were further transmitted into roots and leaves during wheat growth (in vivo and in vitro conditions); Figure 6. This was confirmed in a culture-independent approach. 

**Figure 8:**
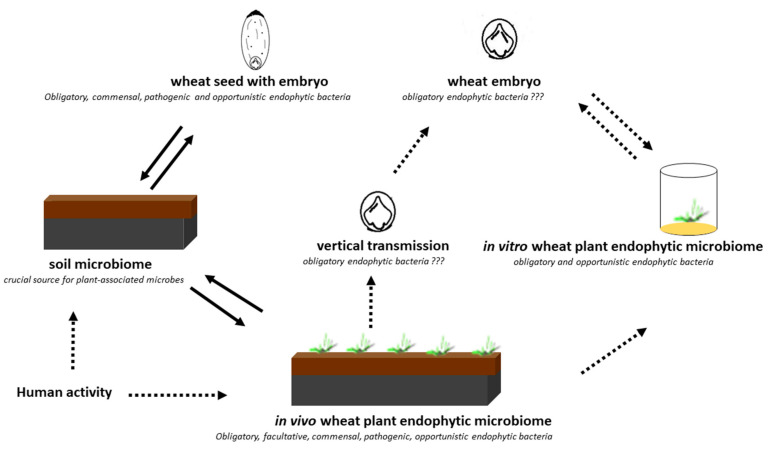
Scheme of the experiment performed in the present study and the potential pathway.

Our results are consistent with those reported by Cope-Selby et al. [20], who suggest that the major source of endophytes in *Miscanthus* is vertical transmission via the seed, presumably supplemented by the ingress of soil bacteria as the plant grows. It was also emphasized that, while studying microbial assemblages within the seeds of different plant species, it is important to understand the location and origin of the microorganisms [7]. Hence, in the present study, we investigated both the embryos and endosperms of selected wheat cultivars. In the culture-independent approach, we confirmed the presence of seven dominant bacterial genera: *Serratia*, *Methanobacterium*, *Streptomyces*, *Lactobacillus*, *Paenibacillus*, *Masilla*, and *Pantoea* with the status of obligatory microorganisms inhabiting wheat seeds in their internal tissues of embryos (definitely lower abundance) and endosperms (Figure 3). *Pantoea* and *Paenibacillus* were found as dominants in the studied seed material, with preference to colonize the endosperms of the studied seeds. The cultured-independent method indicated that some of these bacteria could be transferred from the soil to the seeds. As shown by the literature data [6,21], endophyte species can be transmitted from the vegetative parts of the plant to the seed via the vascular connections from the maternal plant. Endophytes can also be transferred through gametes directly, colonizing the embryo and the endosperm [6,21]. Finally, the vertical transfer of bacteria might also be possible if shoot meristems, which later become reproductive meristems, are colonized, as these eventually give rise to ovules and thus seeds. The transfer of endophytes from mature fruits to seeds has been confirmed as well (Figure 8) [6,21]. 

It is worth mentioning that both genera represent plant growth-promoting bacteria (PGPB). Due to their genetic tractability, ubiquity, and versatility, they are considered as ideal genera for commercial purposes as components of agricultural, medical, and environmental bioproducts [22]. Moreover, the seed-borne *Pantoea* and *Paenibacillus* display anti-fungal properties [23]. A scientific support for our findings is the results published by Coombs and Franco [24], who proved the endophytic colonization of germinating wheat seeds by *Streptomyces* sp. strain EN27. Importantly, the endophytic colonization was observed from a very early stage of plant development with the colonization of the embryo, endosperm, and the emerging radicle [24]. Our study identified the *Streptomyces* species as one of the seven genera inhabiting wheat seeds in their internal embryo tissues. This phenomenon can give grounds for the classification of these strains as obligatory commensal microorganisms of *Triticum aestivum* L. In the context mentioned above, our results are contrary to the findings reported by Robinson et al. [2], who found that the wheat embryo generally exists as a sterile entity and that embryo excision significantly reduced or even eliminated the seed bacterial load. However, it should be mentioned that the wheat seeds in the experiment conducted by Robinson et al. [2] were stored for 2 years at 15 °C before the laboratory analyses. Such a long storage period may have affected their results. 

Our findings can be supported by the results shown by Adams and Kloepper [25], who noted differences in the seed-borne endophytic bacteria between different cotton cultivars, and by Liu et al. [18], who confirmed the same trend in relation to the different maize genotypes. Similarly, Nelson et al. [7] observed that different seeds varied in the microbiota composition, which may be associated with the seed genotypes as well as the environmental conditions in which seeds and seedlings develop. This observation was also confirmed in our experiment involving the seeds of eight different *T. aestivum* and *T. spelta* cultivars (Table 1, Figure 3). 

Kavamura et al. [26] reported that the wheat plants generated from excised embryos had a higher abundance of *Chryseobacterium*, *Dyadobacter*, *Sphingomonas*, *Devosia*, *Caulobacter*, *Phenylobacterium*, *Novosphingobium*, *Rhizobium*, and *Bacillus*, whereas complete seed-derived endosphere samples had a significantly higher abundance of bacteria assigned to *Chitinophaga*, *Pedobacter*, *Flavobacterium*, *Pantoea*, and *Rheinheimera* [26]. In turn, in the case of the bacteria originating from the endosphere of wheat grown in arable soil, only two genera were found to be significantly more abundant in the complete seed-derived wheat plants (*Xanthomonas* and *Paenarthrobacter*) and one genus, i.e., *Chryseobacterium*, was more abundant in the endosphere of the wheat plants generated from excised embryos [26]. The presence of some of the endophytic genera mentioned above was confirmed in our study, i.e., *Chryseobacterium* was found as a shared genus, *Caulobacter* was present in the embryos (Table 1), *Pantoea* inhabited both the endosperms and embryos (Figure 2), *Rhizobium* and *Flavobacterium* were noted in the soil of Hondia, and *Pedobacter* was present in the soil of the Rokosz cultivation (Figure 4 and Figure 5). The fact that bacteria can be present in plant seeds was also recently demonstrated by Glassner et al. [27] in *Cucumismelo* L. seeds, Darrasse et al. [28] in bean seeds, Adams et al. [25] in *Cucurbita pepo* L. seeds, and Sanchez-Lopez et al. [29] in *Crotalaria pumila* L seeds. Furthermore, Sanchez-Lopez et al. [29] provided strong evidence that seed assemblages are similar across several seed generations (in respect to *Crotalaria pumila*). 

It is a known phenomenon that seed-borne endophytes are vertically transmitted from maternal plants to their offspring [1,7,21,30]. This information suggests that the role of seed-borne endophytes is crucial, especially at the early stage of host plant development. Sinnesael et al. [31] showed that plants could not be a sterile entity, as was found in their study of *Burkholderia*-free plants that were able to survive in a sterile in vitro environment, even though the growth of the plants without the endophytes was slower. 

Our studies have shown an interesting situation regarding the presence of endophytes in plant in vitro culture. There are the genera of bacteria that to date have not been identified in seed tissues (Figure 4). The scientific evidence indicates that the seed maturation process not only selects bacteria based on their phenotypic properties, but also that the diversity of the bacterial genera, at least of the cultivable ones, seems to be influenced. The results obtained by Mano and colleagues [32] evidenced the dominance of Gram-negative isolates in the early stages of seed development, but more Gram-positive isolates appeared when the seeds were maturing. In the very early stages, they mainly found *Methylobacterium* sp. and *Sphingomonas* sp., while later *Bacillus* spp. and *Curtobacterium* sp. were more abundant [32]. Furthermore, there is some information in the literature that the phenomena of, for example, the accumulation of starch and the loss of water during the seed maturation process seem to favor endophytes that are tolerant to high osmotic pressure [4,32]. Consequently, the endospore formation can also be an important feature for seed colonizers, as it protects them from changes occurring inside the seed [32,33]. The heatmaps presented in Figure 2 and Figure 4 included: (1) bacteria that were present in at least 10 reads and (2) bacteria that were present in at least three samples (or two samples for the in vitro plants). The results regarding the relative abundance in the seed and plant tissue are presented in Figure 3 and Figure 5 and the Supplementary materials (Tables S3 and S4).

It was found that plant leaves are a diverse microbial habitat, forming an interface between the terrestrial biosphere and solar energy [34]. Moreover, microbial communities may differ between leaves even within a single plant [35]. However, the core traits of the leaves that define microbial habitats are surprisingly sparse [34]. In the current study, wheat root- and leaf-associated microorganisms were evidenced in both in vivo and in vitro experiments (Figure 4 and Figure 5). Greater biodiversity of endophytes was shown in both in vivo and in vitro variants of the rhizospheric soil, roots, and leaves of Hondia rather than in Rokosz. Five of the 31 bacterial genera detected in the soil from the rhizosphere zone, leaves, and roots of Hondia were identified as shared and present in both in vivo and in vitro conditions. These were *Paenibacillus, Propionibacterium*, *Pseudomonas*, *Pedobacter*, and *Staphylococcus* (Figure 6). In contrast, the endophyte biodiversity detected in the Rokosz experiment variants (Figure 7) was limited to 14 genera, among which only one (*Propionibacterium*) was identified in both in vivo and in vitro conditions.

Summarizing, with the use of the culture-independent approach, the seed-borne microbiome colonizing the embryos and endosperms of the selected wheat cultivars was identified. The study can have a practical aspect in the future. For example, Mitter et al. [36] proposed a new approach to modify the plant microbiomes and traits by introducing beneficial bacteria into the progeny seeds at the flowering stage. They described the introduction of *Paraburkholderia phytofirmans* PsJN to the seeds of monocot and dicot plant species and noted the modifications in the seed microbiome structure and growth traits in wheat. Importantly, such studies are a milestone, as they provide information about the beneficial microbes in plants and a means of introducing new traits into plants within one generation without the need of genetic manipulations in plants [36].

## 4. Materials and Methods

### 4.1. Seed Material

The following wheat seeds were studied: winter varieties of *Triticum aestivum* cv. Hondia (H), Wilejka (W), STH, Opcja (O), Tybalt (T), and Euforia (E)and *Triticum spelta* cv. Rokosz (R) and Schwabencorn (Sch) (Figure 8). The seed material was produced by Plant Breeding Strzelce Sp. z o.o. Group IHAR. Prior to DNA isolation, the wheat seeds were surface sterilized following the protocol proposed by Kuźniar et al. [5] and left for overnight imbibition in sterile water at 4 °C. Then, the embryos were carefully and aseptically isolated with the use of a preparative needle and a binocular microscope. The endosperm was sterilized according to the protocol developed by Kuźniar et al. [5].

### 4.2. In Vitro Plant Experiment

Aseptic culture of young seedlings was obtained from the sterile embryos isolated from mature seeds. These seedlings were grown on a medium dedicated for seedling development [37] and containing mineral salts, vitamins, and myo-inositol supplemented with 20 g L^−1^ of sucrose and 7.8 g L^−1^ of agar. The pH of the medium was adjusted to 5.7 prior to sterilization by autoclaving at 121 °C and 103 kPa for 18 min. To obtain sterile seedlings, the mature seeds of the tested varieties were sterilized using the following procedure. In the first step, whole seeds were sterilized for 20 min. in a water solution of sodium hypochlorite (Domestos, Unilever, UK), diluted 1:1 *v*/*v* (commercial bleach:water) and rinsed four times with deionized water for 4–5 min. Then, the whole seeds were placed in deionized water for 2 days (4 °C). This long period of water imbibition was essential to isolate the embryo from the rest of the seed. To separate only the embryo from the whole seed, the isolation was performed under a stereoscopic microscope. In the second step, the isolated embryos were sterilized in a water solution of sodium hypochlorite diluted 1:2 *v*/*v* (commercial bleach:water) and rinsed four times with deionized sterile water for 4–5 min. in sterile conditions. Finally, the sterile embryos were placed in 450 mL glass jars (4 embryos per jar) containing 80 mL of the seedling development medium. Wheat seedlings were grown in a growth chamber at 23 + 1 °C with a 16 h photoperiod (16 h light/8 h darkness). The intensity of light provided by cool white fluorescent tubes was 50 mmol m^−2^ s^−1^. The seedlings were grown in vitro for 3–4 weeks before they were taken for analysis (at stage BBCH 13, Figure 8).

### 4.3. In Vivo Plant Experiment

The studied wheat plants were grown under conventional agricultural practices and conditions of optimum water supply on experimental fields belonging to the Lublin Agricultural Advisory Center (LAAC) in Końskowola, Poland (51°24′33″ N, 22°03′06″ E). Spring barley was the fore crop. The grains were placed at the same depth of 2–3 cm. The seeds were sown on 28 September 2017. Each wheat plant (cultivar Hondia—H; and Rokosz—R) was cleaned from soil in an ultrasonic bath with tap water. Then, the samples of the roots and leaves were separated and surface sterilized. The procedure of sterilization consisted of steps described by Kuźniar et al. [5].

### 4.4. Soil Sampling

Soil materials (according to the Food and Agriculture Organization of the United Nations—FAO classification: *Haplic Podzol*) were collected from the experimental fields belonging to the Lublin Agricultural Advisory Center (LAAC) in Końskowola, Poland (51°24′33″ N, 22°03′06″ E). The samples were taken from the rhizosphere zones of two selected wheat varieties: *T. aestivum* L. cv. Hondia and *T. spelta* L. cv. Rokosz (at wheat growth stage BBCH 13), pooled in an individual plastic bag, and transferred to a portable refrigerator. From each micro-plot (0–20 cm layer), several subsamples were prepared and combined into one (biological replicate). In this way, two biological samples were obtained [38].

### 4.5. DNA Extraction and Next Generation Sequencing (NGS)

The sterilization process performed before the DNA isolation was followed by a crucial step of sterilization quality testing. The asepticity of the materials was confirmed in two strategies: culture methods by plating 100 μL of the last rinsing water onto a solid medium (nutrient agar—BTL sp. z.o.o. Poland, i.e., a universal medium for the growth of a wide variety of bacterial microorganisms) and non-culture PCR methods. The template for the PCR reaction was the last rinsing water. The PCR reaction was performed as described below. Negative controls for PCR were also applied (Supplementary Photo S1) with the assumption that a negative PCR result (and no DNA content in the samples) was considered as successful sterilization.

Triplicate portions of the selected plant materials (after proper sterilization): the roots (K) and leaves (L) (ca. 1 g) of *T. aestivum* L. cv. Hondia and *T. spelta* L. cv. Rokosz were aseptically cut in a laminar chamber, macerated, and homogenized. The embryos and endosperms of the seeds (description in Section 4.1) also were aseptically homogenized with a mortar and pestle. All homogeneous materials (250 mg) were applied for three independent DNA extractions performed with the Power Soil DNA Isolation Kit according to the manufacturer’s instructions (QIAGEN, Hilden, Germany). The kit is designed for the isolation of genomic DNA from environmental samples [39]. Then, the quantity and purity of the DNA were evaluated with a BioSpectrometer^®^ (Eppendorf, Hamburg, Germany). When three DNA samples did not show statistical differences between their purity and quantity, the materials were pooled and mixed well in a single tube as described by Kuźniar et al. [5]. This procedure was described earlier by Soliman et al. (2017) [40]. The authors evidenced that pooling DNA extractions from individual soil samples increased OTU richness. They also referred to the team research conducted by Song et al. (2015) [41], who found that the PCR DNA template amount and soil sample pooling had a significant influence on fungal richness and community composition. All the analyzed DNA samples tested positive for the presence of plant DNA. This information was necessary for the evaluation of successful DNA extraction. Each DNA sample was tested for the presence of plant DNA with primers for the amplification of a highly conserved region of chloroplast DNA (primer 1—5′-AGTTCGAGCCTGATTATCCC-3′ and primer 2—5′-GCATGCCGCCAGCGTTCATC-3′—Phire Plant Direct PCR Kit by Finnzymes). The next step consisted in the PCR reaction, where the total DNA (after pooling triplicate samples) was used as the template DNA. This stage was necessary for the verification of the quality and usefulness of DNA for the NGS sequencing. Universal primers amplifying the variable regions of 16S rRNA: 27F (5′-AGAGTTTGATCATGGCTCAG-3′) and 1492R (5′-TACCTTGTTACGACTT-3′) were applied for the PCR reaction [42,43]. The PCR conditions were as follows: 98 °C for 5 min; 30 cycles of 98 °C for 35 s, 54 °C for 45 s, and 72 °C for 60 s. Moreover, the 5x FIREPol^®^ Master Mix (Soils BioDyne, Tartu, Estonia) was used for the PCR reaction. Finally, the PCR products were run on agarose gel (1%) and visualized with SimplySafe™ (EURx, Gdańsk, Poland). After obtaining positive PCR results (product size 1500 bp; Supplementary Photo S2), the pooled DNA was sequenced by Genomed S.A. (Warsaw, Poland) using the MiSeq 2000 platform (Illumina Inc., San Diego, CA, USA). The metabarcoding analysis were using V3–V4 fragments of the 16S rRNA gene (the primer pair: 341F and 785R). The PCR reactions were performed with Q5 Hot Start High-Fidelity 2X Master Mix according to manufacturer’s recommendations.

The presented results were obtained from the DNA samples that were pooled after the extraction from the independent isolation of three samples.

The data are available under accession number PRJNA547686 (GenBank, NCBI, https://www.ncbi.nlm.nih.gov/sra/?term=PRJNA547686).

### 4.6. Bioinformatic Analysis of Data

Amplicon sequence variants (ASVs) were resolved with DADA2 version 1.14 package [44] and in R version 3.6.0 [45]. Based on the sequence quality plots, the forward and reverse reads were trimmed to 250 and 240 bp, respectively, and the primers and adapter sequences were removed from all reads. The following filtering parameters were used: maxN = 0, maxEE = 3, truncQ = 2. Other parameters were set to default. On average, 126.980 (83%) of reads were obtained after the filtration steps (min = 16,992, max = 206,513, median = 156,267). The error rates were estimated by learn Errors using one million reads. Sequences were dereplicated using derepFastq with default parameters and the exact sequence variants were resolved using DADA. Then, the BimeraDenovo was used to remove chimeric sequences.

Taxonomy was assigned against the latest version of the RDP (modified version 16 available here: http://www2.decipher.codes/Classification/TrainingSets/RDP_v16-mod_March2018.RData) using the IDTAXA Classifier [46]. The resulting taxonomy and read-count tables constructed in DADA2 were appropriately converted and imported into the phyloseq (1.22.3) package [47]. The sequences identified as representing chloroplasts and mitochondria were removed. The sequences from the negative control were subtracted from all samples for subsequent analysis. PERMANOVA (using the Bray–Curtis distance algorithm and 9999 permutations) and alfa- and beta-diversity analyses were carried out in PAST (v. 3.2.5) software [48]. A non-metric multidimensional scaling (NMDS) plot was generated using the Bray–Curtis distance on the OTU table. A heatmap-like diagram was created in R using a heatmap package (v.1.0.12) only for the most abundant genera that were found in at least three different samples and their abundance reached ten or more. The abundance table was then converted into a binary matrix. The distance matrix was calculated using a binary method. Both the Venn diagram and the network-like Venn diagrams were produced in Cytoscape (v.3.7.2) [49]. 

## 5. Conclusions

This study was performed to extend the knowledge of seed-borne endophytic microbiome. It was proven that bacteria colonize both commercially available wheat seeds sown into the soil and those originating from an in vitro experiment.

A greater biodiversity of the seed-borne endophytes was found in the seed endosperms than in the embryos. It was demonstrated that the seed-borne microbiome is dependent on the wheat cultivars. Consequently, it is not possible to conclude that every wheat seed is the same. Moreover, although the embryos of the wheat seeds were inhabited by a rather low number of bacteria, they could not be defined as sterile entities, since (as was evidenced here) the endophytes had an ability to colonize the wheat seed embryos and were further transmitted during wheat growth (i.e., to the roots and/or leaves).

We demonstrated for the first time that seven bacterial genera (*Pantoea*, *Paenibacillus*, *Streptomyces*, *Massilla*, *Lactobacillus*, *Methanobacterium*, *Serratia*) could be classified as obligatory microorganisms colonizing the seeds of the selected *T. aestivum* and *T. spelta* cultivars. The first two genera mentioned above were identified as dominants in the studied seed material. Importantly, we evidenced that the endophytes were present in both the roots and leaves in the in vitro conditions. However, it was proven that the roots of Hondia and Rokosz cv. are more preferable organs for endophytes than the leaves, where their biodiversity was limited. The in vitro experiment also showed that two bacterial genera—*Paenibacillus* and *Propionibacterium*—have a status of obligatory microorganisms inhabiting Rokosz and Hondia cultivars. To sum up, our research clearly showed that wheat seeds (both the endosperm and the embryo) are colonized by endophytic bacteria. Hence, seeds cannot be regarded as sterile niches, because they contain endophytic microbiota. In further research, it will be important to isolate and determine the function of the identified bacterial strains in the growth and subsequent developmental stages of plants. We also evidenced that the community structure of seed-borne endophytes is an individual feature of each wheat cultivar.

## Figures and Tables

**Table 1:** Seed-borne genera identified in the endosperms and embryos of eight wheat cultivars (in alphabetical order).

Endosperms	Embryos	Shared Genera
*Acidovorax*, *Achromobacter*, *Actinoallomurus*, *Actinomyces*, *Alloprevotella*, *Burkholderia*, *Clostridium_IX*, *Curvibacter*, *Dechloromonas*, *Desulfitobacterium*, *Dialister*, *Duganella*, *Ethanoligenens*, *Geobacter*, *Herbaspirillum*, *Hyphomicrobium*, *Longilinea*, *Lysinibacillus*, *Melioribacter*, *Methylobacterium*, *Microbacterium*, *Nitrosomonas*, *Nocardioides*, *Prevotella*, *Roseburia*, *Sanguibacter*, *Smithella*, *Sphingobacterium*, *Sphingomonas*, *Sphingopyxis*, *Solibacillus*, *Subdoligranulum*, *Taibaiella*, *Terribacillus*, *Verrucosispora*	*Anaerococcus*, *Anaeromyxobacter*, *Bergeyella*, *Brevundimonas*, *Caulobacter*, *Ciceribacter*, *Clostridium-sensu-stricto*, *Comamonas*, *Dehalobacter*, *Enhydrobacter*, *Fusobacterium*, *Granulicatella*, *Haemophilus*, *Ignavibacterium*, *Kocuria*, *Melaminivora*, *Paracoccus*, *Soonwooa*, *Solimonas*, *Turicella*	*Chryseobacterium*, *Curtobacterium*, *Lactobacillus*, *Massilia*, *Methanobacterium*, *Neisseria*, *Paenibacillus*, *Pantoea*, *Serratia*, *Streptomyces*

## Supplementary Materials

Supplementary materials can be found at https://www.mdpi.com/1422-0067/21/13/4634/s1.
