# Supplementary material for: New Insight into the Composition of Wheat Seed Microbiota
Source: Int J Mol Sci. 2020 Jun 30;21(13):4634. doi: 10.3390/ijms21134634 (PMC7370184; doi:10.3390/ijms21134634)
Supplement: Supplementary file 1 [file ijms-21-04634-s001.zip › supplementary/Photo S1 negative.docx]

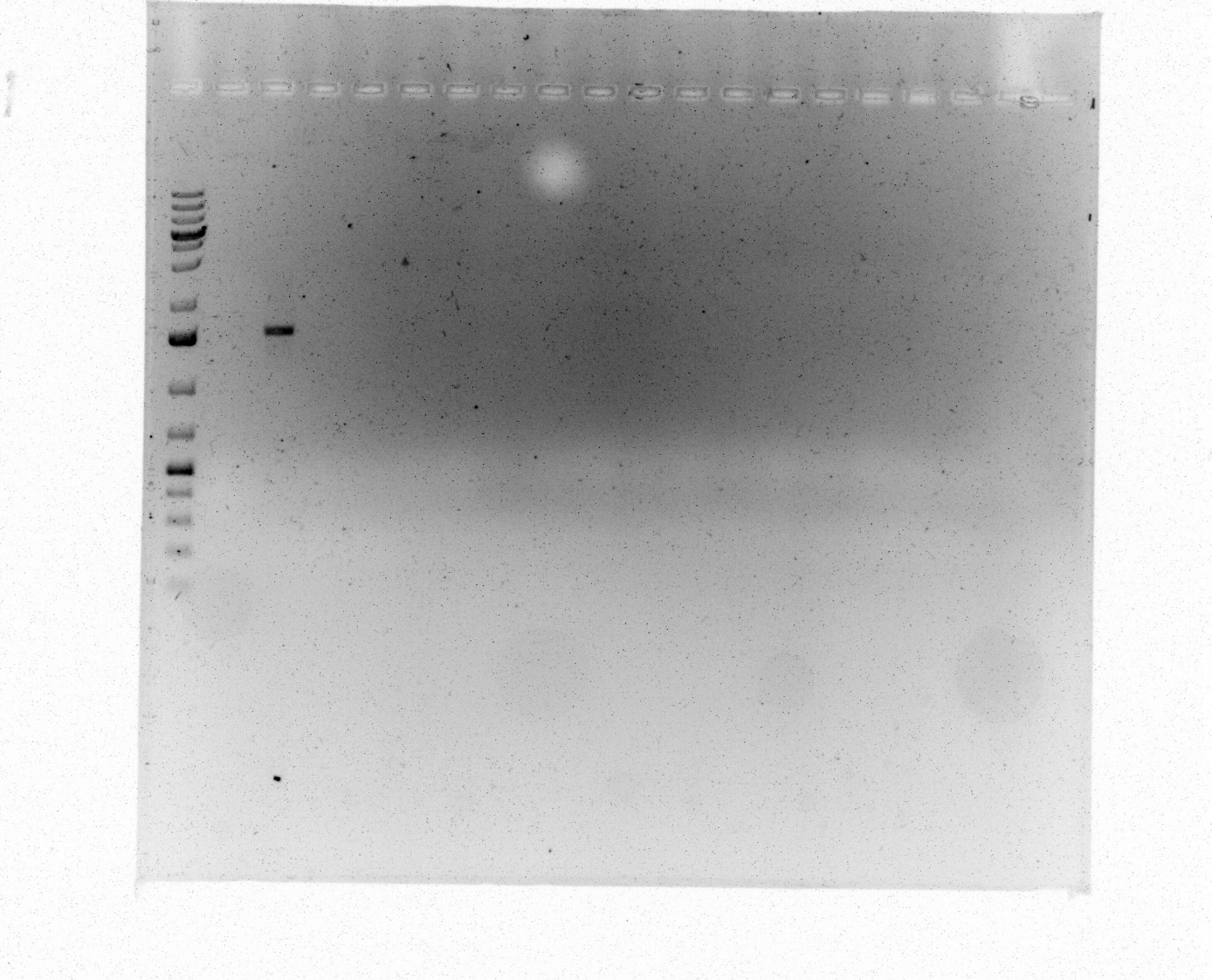


A

**1 2 3 4 5 6 7 8 9 10 11 12 13 14 15 16 17 18**


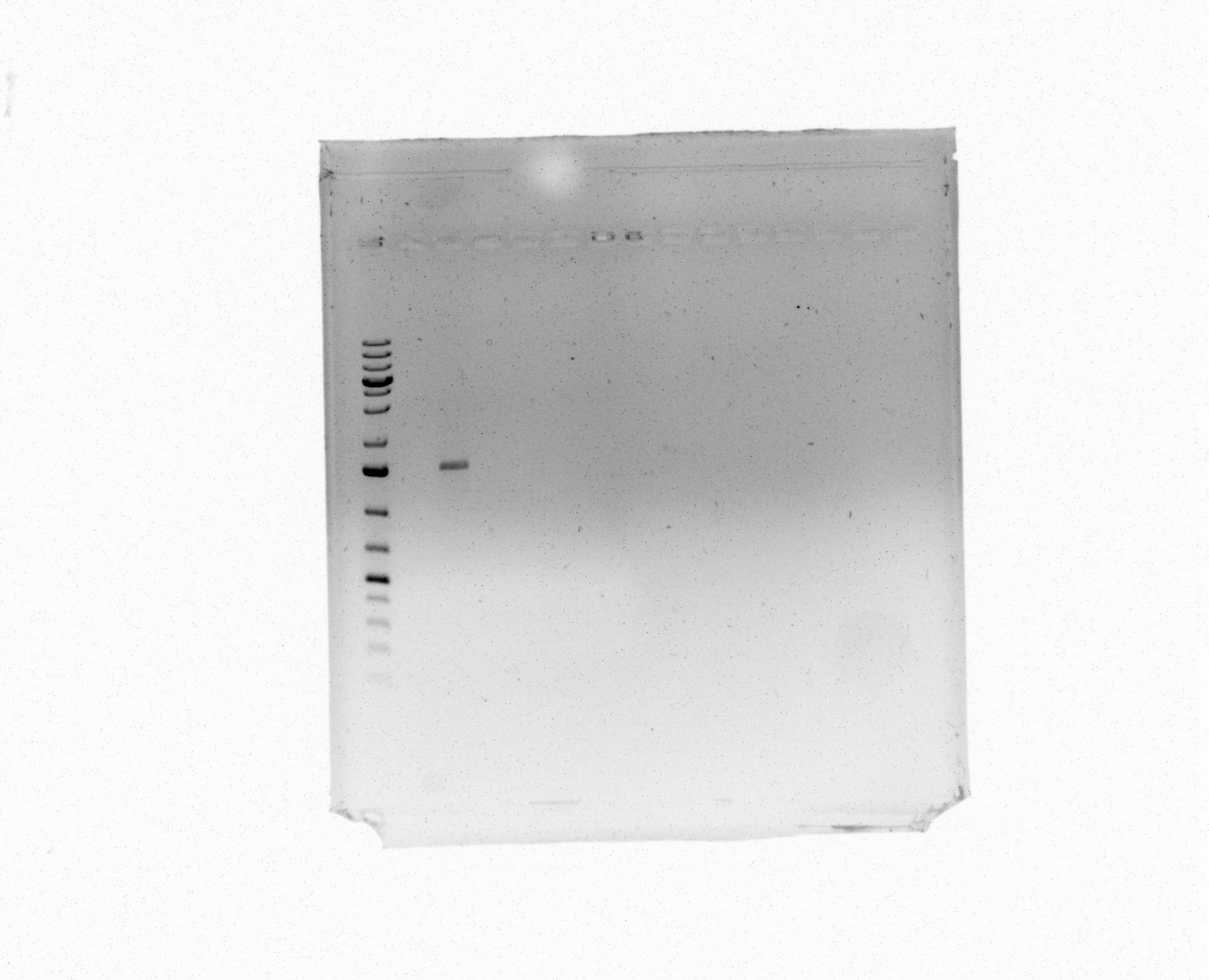


B

**Photo**  Agarose gel electrophoresis of PCR products treated as probes. The following lines are marked as follows:

A

1. Marker of 1kb ladder plus
2. Positive control for PCR reaction
3. Negative control for PCR reaction
4. The last water after sterilization of Hondia – endosperm
5. The last water after sterilization of Wilejka - endosperm
6. The last water after sterilization of STS - endosperm
7. The last water after sterilization of Opcja - endosperm
8. The last water after sterilization of Tybalt - endosperm
9. The last water after sterilization of Euforia - endosperm
10. The last water after sterilization of Rokosz - endosperm
11. The last water after sterilization of Schwabencorn - endosperm
12. The last water after sterilization of Hondia – embryo
13. The last water after sterilization of Wilejka - embryo
14. The last water after sterilization of STS - embryo
15. The last water after sterilization of Opcja - embryo
16. The last water after sterilization of Tybalt - embryo
17. The last water after sterilization of Euforia – embryo
18. The last water after sterilization of Rokosz - embryo

B

1. Marker of 1kb ladder plus
2. Positive control for PCR reaction
3. Negative control for PCR reaction
4. The last water after sterilization of Hondia – leaves
5. The last water after sterilization of Hondia – roots
6. The last water after sterilization of Rokosz - leaves
7. The last water after sterilization of Rokosz - roots
