# Supplementary material for: New Insight into the Composition of Wheat Seed Microbiota
Source: Int J Mol Sci. 2020 Jun 30;21(13):4634. doi: 10.3390/ijms21134634 (PMC7370184; doi:10.3390/ijms21134634)
Supplement: Supplementary file 1 [file ijms-21-04634-s001.zip › supplementary/Photo S2.docx]

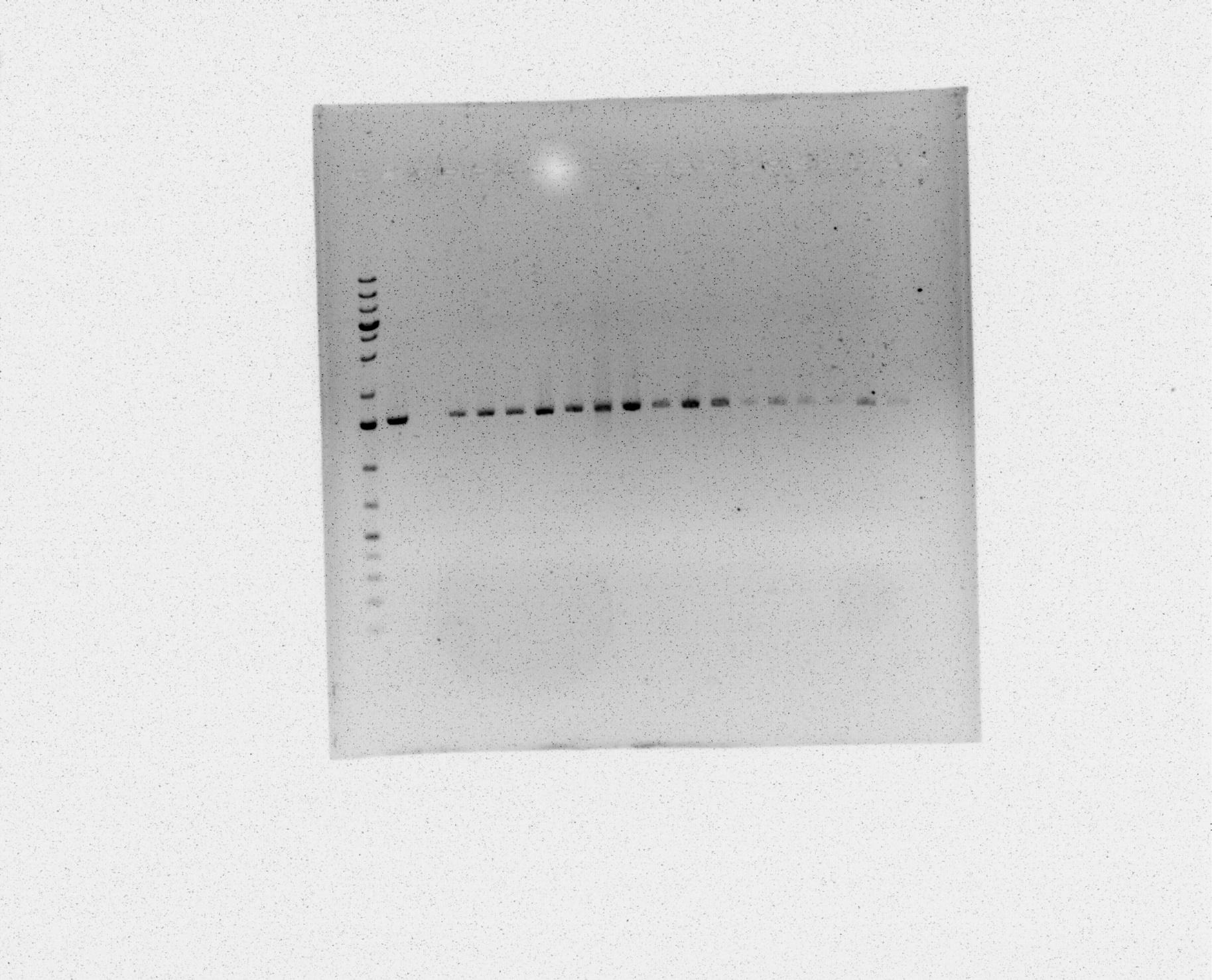


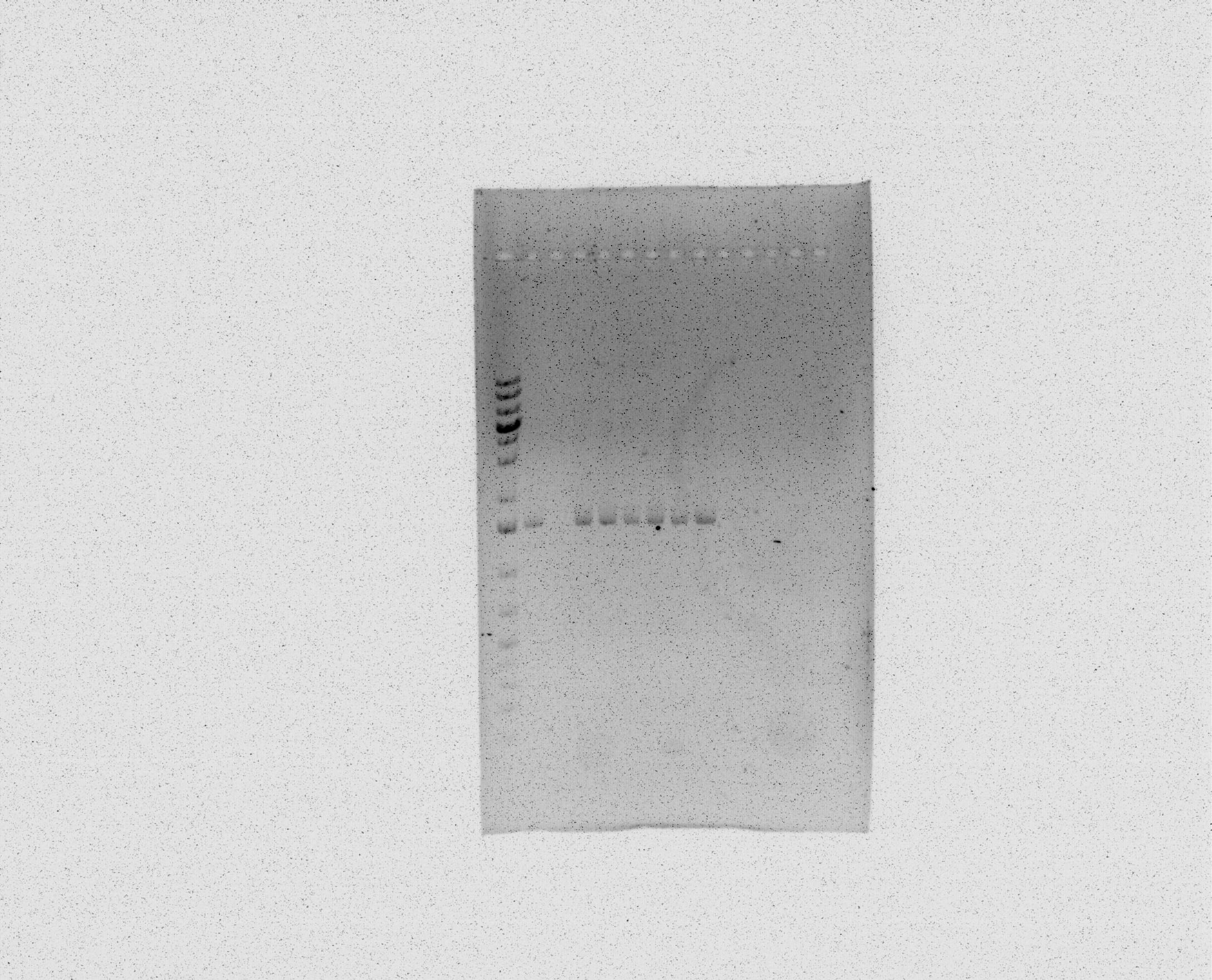


**Photo …..**Agarose gel electrophoresis of PCR products treated as probes. The following lines are marked as follows:

**A**

1. Marker of 1kb ladder plus
2. Positive control for PCR reaction
3. Negative control for PCR reaction
4. DNA template of Hondia – endosperm
5. DNA template of Wilejka - endosperm
6. DNA template of STS - endosperm
7. DNA template of Opcja - endosperm
8. DNA template of Tybalt - endosperm
9. DNA template of Euforia - endosperm
10. DNA template of Rokosz - endosperm
11. DNA template of Schwabencorn - endosperm
12. DNA template of Hondia – embryo
13. DNA template of Wilejka - embryo
14. DNA template of STS - embryo
15. DNA template of Opcja - embryo
16. DNA template of Tybalt - embryo
17. DNA template of Euforia – embryo
18. DNA template of Rokosz - embryo

**B**

1. Marker of 1kb ladder plus
2. Positive control for PCR reaction
3. Negative control for PCR reaction
4. DNA template of Hondia – leaves
5. DNA template of Hondia – roots
6. DNA template of Rokosz - leaves
7. DNA template of Rokosz - roots
